# Electronic–photonic interplay in nitrogen-doped MXene quantum dots: mechanistic insights into dual-mode and multiplexed sensing

**DOI:** 10.1039/d6ra02859d

**Published:** 2026-05-20

**Authors:** Kamel A. Saleh, Wael Waleed Mustafa, Rekha M. M., Subhashree Ray, Talal Aziz Qassem, T. Krithiga, Renu Sharma, Prakhar Tomar, Shayan Mahmoodi

**Affiliations:** a Faculty of Allied Medical Sciences, Hourani Center for Applied Scientific Research, Al-Ahliyya Amman University Amman Jordan; b College of Pharmacy, Department of Pharmaceutical Sciences, AL-Turath University Baghdad Iraq; c Department of Chemistry and Biochemistry, School of Sciences, JAIN (Deemed to be University) Bangalore Karnataka India; d Department of Biochemistry, IMS and SUM Hospital, Siksha ‘O’ Anusandhan Bhubaneswar Odisha-751003 India; e Department of Medical Laboratory Techniques, College of Health and Medical Technology, Alnoor University Mosul Iraq; f Department of Chemistry, Sathyabama Institute of Science and Technology Chennai Tamil Nadu India; g Department of Chemistry, University Institute of Sciences, Chandigarh University Mohali Punjab India; h Centre for Research Impact & Outcome, Chitkara University Institute of Engineering and Technology, Chitkara University Rajpura 140401 Punjab India; i Young Researchers and Elite Club, Islamic Azad University Tehran Branch Tehran Iran sh.mahmoodiacademic@gmail.com

## Abstract

Nitrogen-doped MXene quantum dots (N-MQDs) are emerging nanomaterials with great potential for dual-mode and multiplexed sensing owing to their tunable photoluminescence, high electrical conductivity, and chemically active surfaces. In this work, we aim to clarify the fundamental mechanisms governing the coexistence and coupling of optical and electronic responses in N-MQDs and to provide design principles for their application in sensing platforms. Specifically, the study focuses on how nitrogen doping and surface coordination modify the electronic structure, density of states, and Fermi level alignment, thereby influencing radiative emission, charge transport, and analyte interaction. The sensing behavior is interpreted through the balance between localized dopant-induced states and the delocalized MXene conduction network, which enables dual signal generation with reduced mutual interference. The phenomena are discussed in terms of photoluminescence response, carrier dynamics, surface-state effects, and charge-transfer processes, while the roles of dopant configuration and surface terminations in controlling signal orthogonality and crosstalk suppression are highlighted. Overall, this work presents a mechanistic framework for the rational design of N-MQD-based dual-mode sensing systems for environmental, chemical, and biomedical applications.

## Abstract 2

Nitrogen-doped MXene quantum dots (N-MQDs) enable sensitive dual-mode and multiplex sensing *via* fluorescence quenching, FRET, IFE, and coordination interactions for environmental and biomedical applications.

## Graphical abstract

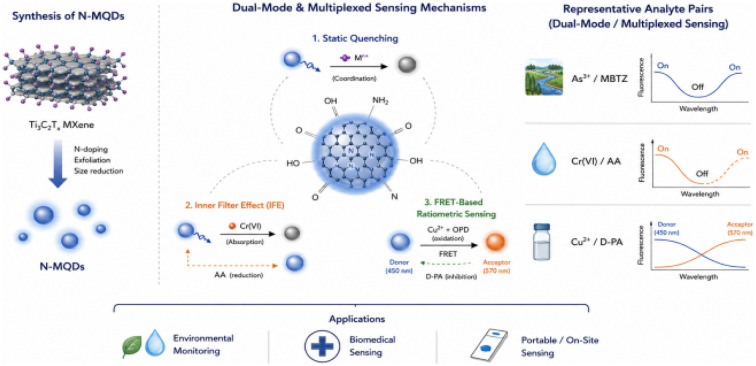

## 1. Introduction

The rapid advancement of chemical and biological sensing technologies has created an increasing demand for multifunctional platforms capable of delivering reliable, sensitive, and information-rich outputs under complex and variable conditions.^1,2^ Conventional single-mode sensors, relying solely on optical or electrochemical signals, often suffer from limited robustness, susceptibility to environmental interference, and an inability to cross-validate analytical results.^3,4^ In response to these challenges, dual-mode and multiplexed sensing strategies have emerged as powerful alternatives, offering complementary signal channels that enhance accuracy, reliability, and analytical depth.^5,6^ Central to the success of such systems is the development of nanomaterials capable of supporting coordinated yet non-redundant optical and electronic responses within a single architecture.^7–10^

Quantum dots (QDs) have long attracted attention in sensing applications due to their size-dependent electronic structure, tunable photoluminescence, and high surface-to-volume ratio.^11,12^ However, many conventional QDs exhibit intrinsically insulating or semiconducting behavior, which limits their effectiveness in electronic or electrochemical transduction. Conversely, materials with excellent electrical conductivity often lack efficient or stable optical emission.^13,14^ Bridging this functional divide remains a fundamental challenge in the design of true dual-mode sensing materials, where optical and electronic signals must coexist without mutual suppression.^15^

MXenes, a rapidly expanding family of two-dimensional transition metal carbides and nitrides, generally described by the formula M_*n*+1_X_*n*_T_*x*_ (where M represents an early transition metal such as Ti, V, or Nb; X is carbon and/or nitrogen; *n* = 1–4; and T_*x*_ denotes surface termination groups such as –O, –OH, or –F), offer a compelling solution to this challenge. Their metallic or semi-metallic conductivity, hydrophilic surfaces, and chemical versatility make them attractive for electrochemical and electronic applications.

Their metallic or semi-metallic conductivity, hydrophilic surfaces, and chemical versatility make them attractive for electrochemical and electronic applications.^16,17^ When confined to the quantum dot regime, MXenes acquire additional photophysical characteristics, including size-dependent emission and enhanced surface reactivity. MXene quantum dots (MQDs) thus combine conductive frameworks with emerging optical functionality, positioning them as promising candidates for multimodal sensing platforms.^18–20^

Among various modification strategies, heteroatom doping—particularly nitrogen doping—has proven highly effective in tailoring the electronic and photonic properties of MQDs. Nitrogen incorporation introduces localized electronic states, alters charge distribution, and modifies band structure and density of states near the Fermi level. These effects not only enhance photoluminescence efficiency and wavelength tunability but also preserve or even improve charge transport pathways. As a result, nitrogen-doped MXene quantum dots (N-MQDs) have demonstrated superior performance in a wide range of sensing applications, including the detection of metal ions, metalloids, and small organic molecules.^21–23^

Despite the growing number of experimental demonstrations, the fundamental mechanisms enabling the simultaneous generation of optical and electronic signals in N-MQDs remain insufficiently unified. Many studies report dual-mode behavior phenomenologically, without addressing how localized emissive states and delocalized conductive networks coexist and interact at the electronic level. In particular, the concept of electronic–photonic interplay—referring to the coordinated but not necessarily coherent relationship between charge transport processes and photophysical transitions—has not been systematically articulated.^21,24^ Without such mechanistic understanding, rational design of next-generation dual-mode sensing materials remains largely empirical.

In N-MQDs, the electronic–photonic interplay arises from a delicate balance between localized dopant-induced states responsible for radiative emission and the intrinsic MXene conduction network that supports efficient charge transfer. Nitrogen-related states can act as emissive centers or transient carrier traps, extending excited-state lifetimes while maintaining accessibility for electronic transduction.^25,26^ At the same time, surface functional groups, defect landscapes, and dopant configurations regulate carrier recombination pathways, ensuring that radiative and nonradiative processes do not competitively suppress one another. This semi-decoupled yet cooperative relationship enables signal orthogonality, a defining requirement for reliable dual-mode and multiplexed sensing.^21,27^

Structural and surface-state engineering further modulate this interplay. Defect distribution, surface termination programming, interface architecture with electrodes or substrates, and spatial compartmentalization collectively influence how signals are generated, routed, and extracted. Rather than acting as passive features, these parameters function as active design variables that govern crosstalk suppression, signal robustness, and environmental tolerance. Understanding their mechanistic roles is therefore essential for translating nanoscale material properties into predictable macroscopic sensing performance.^28–30^

In this work, we present a comprehensive mechanistic framework describing the electronic–photonic interplay in N-MQDs and its role in enabling dual-mode and multiplexed sensing. By integrating insights from electronic structure modulation, carrier dynamics, surface chemistry, and structural engineering, we establish clear design principles that link fundamental material behavior to practical sensing outcomes. This perspective not only rationalizes existing experimental observations but also provides a roadmap for the development of advanced N-MQD-based sensing platforms for environmental, chemical, and biomedical applications.

**Fig. 1:**
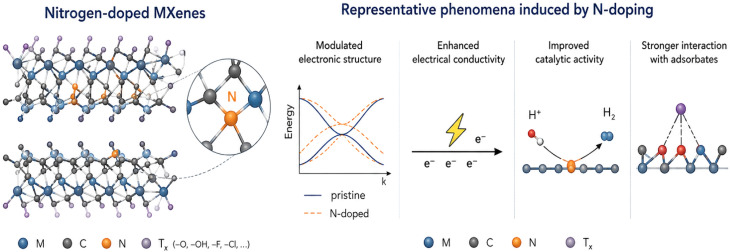
Schematic illustration nitrogen-doped MXenes and the representative effects induced by N incorporation, including modulation of electronic structure, enhanced electrical conductivity, improved catalytic activity, and stronger interaction with adsorbates.

MXenes are a rapidly emerging family of two-dimensional transition metal carbides and/or nitrides with a general formula of M_*n*+1_X_*n*_T_*x*_, where M represents an early transition metal, X denotes carbon and/or nitrogen, and T_*x*_ corresponds to surface termination groups such as –O, –OH, and –F. Owing to their layered structure and tunable surface chemistry, MXenes exhibit remarkable electronic, catalytic, and adsorption properties. Recently, nitrogen doping has attracted considerable attention as an effective strategy to further tailor the physicochemical characteristics of MXenes. Nitrogen incorporation can modify the electronic structure, improve conductivity, enhance catalytic performance, and strengthen interactions with adsorbed species (Fig. 1).

## 2. Electronic–photonic interplay in nitrogen-doped MXene quantum dots

### 2.1. Nitrogen-induced band structure modulation and its role in dual-mode responses

Nitrogen doping profoundly alters the electronic band structure of MQDs, laying the foundation for their simultaneous optical and electronic responses. In pristine MXene QDs, the electronic structure is dominated by metallic or semi-metallic behavior with a high density of states near the Fermi level, which favors charge transport but limits radiative recombination. The introduction of nitrogen heteroatoms disrupts this balance by inserting localized electronic states within the bandgap or near band edges. These dopant-induced states act as intermediate energy levels that mediate both electron transfer and photon emission.^18,31^

From a theoretical perspective, nitrogen atoms—owing to their higher electronegativity compared with carbon—induce charge redistribution within the MXene lattice. This redistribution modifies band curvature and effective carrier mass, affecting both conductivity and optical absorption. Importantly, the coexistence of delocalized metallic states and localized dopant states creates a hybrid electronic environment. Such an environment is particularly conducive to dual-mode transduction, as electronic conduction pathways and radiative transitions can coexist without mutually quenching each other.^24,26^

**Fig. 2:**
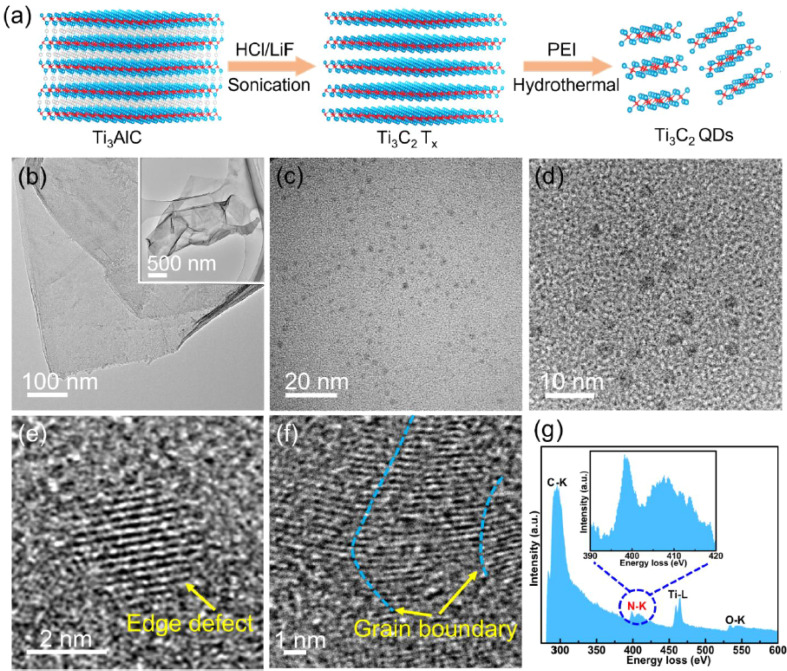
Synthesis and structural characterization of nitrogen-doped Ti_3_C_2_T_*x*_ quantum dots illustrating quantum confinement and nitrogen-induced electronic structure modulation. (a) Schematic of the multi-step process from Ti_3_AlC_2_ to PEI-assisted hydrothermal synthesis of ∼3 nm N-MQDs. (b) TEM of pristine multilayered Ti_3_C_2_T_*x*_ nanosheets. (c) and (d) Low- and high-magnification TEM of uniform ∼3 nm QDs. (e) and (f) HR-TEM revealing (105) lattice fringes (0.215 nm) and abundant edge/grain boundary defects. (g) EELS spectrum confirming N incorporation (N–K edge). Adapted with permission from ref. 31. © 2024 Elsevier B.V.

Fig. 2 illustrates the structural and physicochemical characteristics of the synthesized N-MQDs and their implications for dual-mode sensing performance. Panel (a) confirms the successful formation of nanoscale quantum dots with relatively uniform lateral dimensions. However, beyond morphological verification, the size distribution critically influences quantum confinement and defect density, which in turn modulate band structure and fluorescence behavior. Variations in particle size may contribute to emission heterogeneity and should be considered when evaluating batch-to-batch reproducibility.

Panel (b) presents high-resolution structural features, indicating lattice fringes consistent with preserved MXene crystallinity after quantum confinement. While this suggests structural integrity, partial disorder and edge reconstruction—common in etched MXene derivatives—may introduce localized electronic states that significantly affect carrier recombination dynamics. Panel (c) (XPS analysis) demonstrates the presence of multiple nitrogen configurations. Rather than merely confirming doping, these results imply redistribution of electron density and modification of the density of states (DOS) near the Fermi level. However, quantitative deconvolution uncertainties and surface sensitivity of XPS may limit precise correlation between nitrogen configuration ratios and optical performance. Panel (d) correlates optical absorption and photoluminescence features with defect-mediated transitions. The emission profile suggests combined band-edge and surface-state contributions, yet the relative dominance of each pathway requires complementary time-resolved measurements for conclusive interpretation.

### 2.2. Charge carrier dynamics governing radiative and nonradiative signal pathways

Dual-mode behavior in N-doped MQDs ultimately depends on how charge carriers are generated, transported, and dissipated. Upon external stimulation, such as electrical bias or photoexcitation, electrons and holes populate excited states and must choose between radiative recombination, nonradiative relaxation, or interfacial charge transfer. Nitrogen doping plays a decisive role in regulating these competing pathways.^21,26^ Dopant-induced trap states can prolong carrier lifetimes by temporarily localizing electrons or holes. Extended lifetimes increase the probability of radiative recombination, enhancing photonic output, while simultaneously allowing sufficient time for carriers to participate in electrochemical or electronic transduction processes. This dual accessibility of carriers is a defining feature of materials capable of multimodal signal generation.^32^

Additional experimental support for the proposed electronic–photonic coupling mechanism in N-doped MQDs has been obtained through complementary spectroscopic and electrochemical characterization techniques. Time-resolved photoluminescence (TRPL) measurements have frequently been employed to probe carrier recombination dynamics and excited-state lifetimes. Variations in fluorescence decay profiles after nitrogen incorporation indicate the coexistence of multiple relaxation pathways involving both localized emissive states and interfacial charge-transfer processes. In several reports, shortened or multicomponent PL lifetimes were correlated with enhanced nonradiative electron-transfer kinetics while maintaining measurable radiative emission, suggesting partial independence between optical and electronic signal channels. Electrochemical impedance spectroscopy (EIS) has further demonstrated reduced charge-transfer resistance and improved interfacial conductivity in N-modified MQDs, supporting efficient carrier transport through conductive MXene networks.^32–34^ In addition, transient absorption spectroscopy (TAS) has been utilized in selected studies to monitor ultrafast carrier relaxation and photoinduced charge separation processes associated with nitrogen-derived intermediate electronic states. Collectively, these findings support the coexistence of coupled yet partially decoupled photonic and electronic pathways within N-MQDs, providing mechanistic evidence for their stable dual-mode transduction behavior under a single excitation stimulus.

**Fig. 3:**
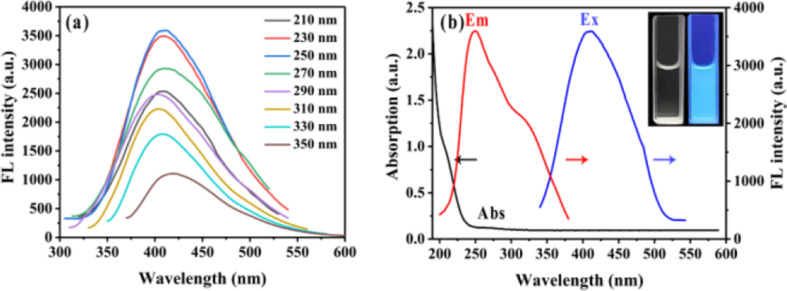
Excitation-wavelength-dependent photoluminescence of nitrogen-doped Ti_3_C_2_T_*x*_ quantum dots demonstrating surface-state-dominated emission arising from nitrogen-induced localized electronic states within the effective bandgap. (a) PL spectra (*λ*_ex_ = 210–350 nm) showing progressive red-shift and maximum intensity at 250 nm. (b) UV-vis absorption (black), emission at 410 nm (red), excitation spectrum (blue), with inset photographs of intense blue fluorescence under 365 nm UV. PLQY = 13.55%. Adapted with permission from ref. 32. © 2021 American Chemical Society.

Fig. 3 provides photophysical evidence for nitrogen-induced electronic restructuring in Ti_3_C_2_T_*x*_ quantum dots (MQDs), highlighting how quantum confinement and surface functionalization alter the originally metallic electronic character of MXene.

Panel (a) presents excitation-dependent photoluminescence spectra of the N-MQDs dispersed in water, recorded with excitation wavelengths ranging from 210 to 350 nm. The systematic red-shift of the emission maximum with increasing excitation wavelength, together with the strongest emission at *λ*_ex_ = 250 nm, indicates that the luminescence is dominated by surface or defect states rather than intrinsic band-edge recombination. Such excitation-dependent emission suggests a heterogeneous distribution of emissive centers, likely originating from nitrogen-induced defect states, edge functional groups, and structural disorder introduced during the quantum-dot formation process. While this behavior supports the hypothesis that nitrogen incorporation perturbs the density of states and enables radiative recombination pathways, the broad distribution of emissive states also implies potential variability in emission characteristics, which may influence reproducibility across different synthesis batches.

Panel (b) further reflects the modified electronic structure through combined UV-vis absorption, excitation, and emission spectra. The absorption feature near 210 nm and the emission centered around 410 nm under 250 nm excitation produce a large Stokes shift (>150 nm), indicating that excited carriers undergo significant relaxation through intermediate surface-derived states before recombination. This observation is consistent with the presence of defect-mediated transitions generated by nitrogen doping and surface termination. However, although the measured photoluminescence quantum yield (13.55%) confirms the emergence of radiative pathways absent in pristine metallic MXene, the exact contribution of different nitrogen configurations and surface terminations to the emissive states cannot be resolved solely from steady-state spectroscopy. Complementary time-resolved measurements would therefore be necessary to quantitatively elucidate carrier trapping and recombination dynamics.

The dual-mode functionality observed in N-MQDs originates from the regulated dynamics of charge carriers between radiative emission, nonradiative relaxation, and interfacial charge-transfer pathways. Nitrogen incorporation introduces localized electronic trap states that extend carrier lifetimes and provide intermediate energy levels capable of mediating both photonic and electronic processes. These dopant-induced states temporarily localize excited carriers, increasing the probability of radiative recombination while still maintaining sufficient mobility for participation in electrochemical or electronic transduction. Importantly, the density and energetic depth of these trap states must remain within an optimal range; excessive trapping promotes phonon-assisted nonradiative decay, whereas insufficient trapping limits radiative efficiency and signal generation. The dual-mode response therefore arises from a balanced carrier environment in which localized emissive centers coexist with the delocalized conductive network of the MXene framework. This balance ensures that optical emission and electronic conduction operate cooperatively rather than competitively.^30–33^ Looking forward, precise engineering of dopant configurations, defect distributions, and surface terminations will be essential for tuning carrier lifetimes and minimizing nonradiative losses, thereby enabling more robust and predictable dual-mode sensing performance in future N-MQD-based platforms.

In dual-mode sensing systems based on N-MQDs, the concept of signal orthogonality refers to the degree of functional independence between optical and electrical output channels when responding to the same external stimulus. Ideally, variations in photoluminescence intensity or spectral features should not significantly interfere with electrical responses such as current, resistance, or impedance, and *vice versa*. In the literature, this orthogonality is typically evaluated using semi-quantitative metrics including signal correlation coefficients between optical and electrical responses, crosstalk percentage between detection channels, and the preservation of individual signal-to-noise ratios (SNRs) during simultaneous measurements. Additional indicators include the retention of sensitivity and detection limits in each channel when both outputs are monitored concurrently. While a universally standardized quantitative framework has not yet been established for N-MQD systems, several MXene- and quantum-dot-based multiplexed sensing platforms demonstrate relatively low signal interference due to partial decoupling between photonic emission processes and charge-transfer pathways within conductive MXene networks. These characteristics enable reliable dual-mode signal transduction and provide a practical strategy for improving sensing reliability through cross-validation between independent detection channels.

### 2.3. Electronic density of states modulation and its impact on signal accessibility

One of the most critical electronic consequences of nitrogen doping is the modification of the density of states (DOS) near the Fermi level. In MQDs, the DOS determines how readily carriers participate in charge transfer or optical transitions. Nitrogen incorporation introduces asymmetric DOS features that redistribute electronic populations and realign the Fermi level relative to conduction and valence band edges.^35,36^ From a conceptual standpoint, DOS engineering decouples optical intensity from purely structural factors and links it directly to electronic occupation statistics.^22,37^ Nitrogen-doped MXene QDs thus behave as electronically adaptive emitters rather than fixed photoluminescent materials. This adaptability provides a theoretical explanation for their versatility in dual-mode platforms, independent of specific sensing configurations.

**Fig. 4:**
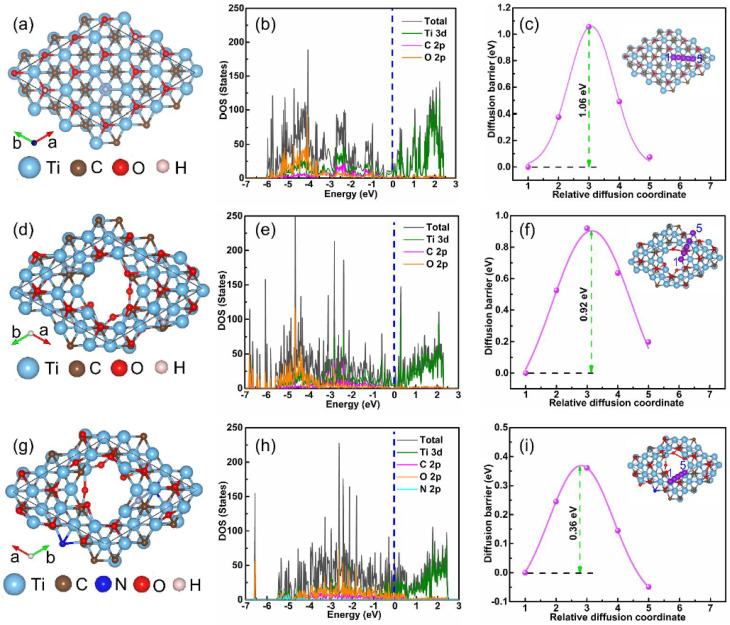
DFT-computed electronic structure modulation induced by nitrogen doping in Ti_3_C_2_T_*x*_ materials. (a, d, g) Optimized atomic structures of pristine O-terminated nanosheets, bare MQDs, and N-doped MQDs. (b, e, h) Total and partial density of states (TDOS/PDOS) showing increased DOS near the Fermi level and N 2p contributions in N-MQDs. (c, f, i) K^+^ diffusion energy barriers (1.06 eV → 0.92 eV → 0.36 eV) with migration paths (insets), demonstrating improved carrier accessibility *via* DOS broadening and Fermi-level realignment. Adapted with permission from ref. 31. © 2024 Elsevier B.V.

Fig. 4 presents density functional theory (DFT) calculations that provide mechanistic insight into how nitrogen incorporation modifies the electronic structure of Ti_3_C_2_T_*x*_ quantum dots and influences carrier accessibility relevant to dual-mode sensing. Rather than only confirming structural modification, these calculations help establish a structure–property relationship linking atomic-scale changes to electronic transport and optical activity.

Panels (a), (d), and (g) illustrate the optimized structures of O-terminated Ti_3_C_2_T_*x*_ nanosheets, bare MQDs, and nitrogen-doped MQDs (N-MQDs), respectively. The transition from extended 2D sheets to confined 0D quantum dots introduces edge reconstruction, under-coordinated atoms, and increased surface area, which already perturb the electronic structure. Nitrogen incorporation further alters the local coordination environment through substitutional doping and surface functionalization, indicating that the electronic behavior of N-MQDs arises from the combined effects of quantum confinement and dopant-induced electronic perturbation. However, the models represent idealized configurations, whereas experimentally synthesized MQDs likely exhibit greater structural heterogeneity and mixed surface terminations. The total and partial density of states (TDOS/PDOS) in panels (b), (e), and (h) reveal a progressive redistribution of electronic states near the Fermi level. Pristine nanosheets display metallic characteristics dominated by Ti 3d orbitals, while MQD formation introduces additional localized states associated with edges and defects. In N-MQDs, nitrogen-derived orbitals contribute to asymmetric DOS broadening and an increased density of states around the Fermi level. This redistribution suggests enhanced electronic accessibility and improved charge transfer capability, although the relative contributions of different nitrogen configurations cannot be fully distinguished from these calculations alone. Panels (c), (f), and (i) compare K^+^ diffusion barriers, showing a substantial decrease from 1.06 eV in nanosheets to 0.36 eV in N-MQDs. This reduction indicates that nitrogen-induced electronic redistribution facilitates ion migration by modifying surface adsorption energetics. Nonetheless, these simulations neglect solvent and electrochemical interface effects, which may influence ion transport behavior under experimental sensing conditions.

### 2.4. Interplay between localized dopant states and delocalized MXene conduction networks

Dual-mode sensing requires communication between two fundamentally different electronic regimes: localized states responsible for light emission and delocalized states enabling charge transport.^38,39^ N-MQDs uniquely accommodate this requirement through intrinsic coupling between dopant-induced localized orbitals and the metallic conduction network of the MXene framework. Localized nitrogen-related states act as emissive centers, while the underlying MXene lattice preserves high electrical conductivity. The coupling between these states allows excited carriers to shuttle between emission and transport pathways without complete energy loss. This hybridization is essential; without it, optical and electronic signals would originate from isolated substructures and fail to respond coherently to a single stimulus.^40,41^

The strength of this coupling depends on orbital overlap, spatial proximity, and electronic symmetry. Nitrogen atoms positioned within conductive domains facilitate stronger coupling than those isolated at surface terminations. When coupling is optimized, electrons excited into localized states can relax radiatively while maintaining connectivity to the conduction network, enabling synchronous signal generation. This framework shifts the interpretation of N-doped MXene QDs from simple luminescent nanomaterials to electronically integrated systems.^36,42^ Dual-mode transduction is thus not an additive property but an emergent phenomenon arising from coherent electronic coupling across length scales. Recognizing this coupling mechanism helps rationalize why certain MXene QDs sustain dual-mode outputs while others fail, even with similar compositions.

### 2.5. Energetic decoupling and its role in achieving signal orthogonality

A crucial but often overlooked requirement for effective dual-mode sensing is energetic decoupling between signal channels. If optical and electronic processes compete for the same energetic states, one signal inevitably suppresses the other. N-MQDs overcome this limitation by distributing photonic and electronic processes across partially independent energy landscapes. Through selective state localization, radiative transitions are confined to energy levels that do not fully overlap with those dominating charge transports. This energetic separation ensures signal orthogonality, meaning that changes in one mode do not trivially mirror changes in the other. Orthogonality is essential for dual-mode systems because it allows cross-validation rather than redundancy.^43,44^

Energetic decoupling is not absolute isolation; weak coupling remains necessary for correlated responses. Instead, the system operates in a semi-decoupled regime where interaction exists without dominance. Nitrogen doping enables this regime by introducing states with intermediate coupling strengths that act as bridges rather than bottlenecks. From a theoretical standpoint, this balance defines the operational window of dual-mode transduction. Outside this window, materials revert to single-mode dominance. Thus, energetic decoupling is not merely advantageous but foundational. It provides a unifying principle explaining how N-MQDs maintain independent yet cooperative optical and electronic responses, a defining hallmark of true dual-mode sensing materials.^33,45^

Taken together, the mechanistic picture presented in this section should be understood as a literature-supported interpretive framework rather than a claim of fully resolved atomistic certainty for every N-MQD system. Experimental observations such as excitation-dependent photoluminescence, measurable quantum yield, XPS-confirmed nitrogen incorporation, and preserved electrical conductivity collectively support the coexistence of localized emissive states and delocalized transport pathways. In parallel, DFT-based analyses reported for related Ti_3_C_2_T_*x*_ quantum dot systems provide consistent evidence that nitrogen incorporation redistributes charge density, perturbs the density of states near the Fermi level, and lowers kinetic barriers for carrier or ion transport.^26–30^ These combined results substantiate the central proposition that nitrogen doping does not simply increase defect density, but reorganizes the electronic landscape into a semi-decoupled architecture capable of sustaining both radiative and electronic outputs. At the same time, the exact contribution of specific nitrogen configurations, surface terminations, and defect populations remains system-dependent and may vary with synthesis route. Therefore, the framework proposed here is intended to distinguish established trends from emerging mechanistic hypotheses and to provide a rational basis for future experimental validation and device optimization.

This energetic semi-decoupling also helps clarify the distinctive position of MQDs relative to carbon dots (CDs) in dual-mode sensing. While CDs are highly attractive fluorescent nanomaterials because of their low toxicity, facile synthesis, and rich surface-state photophysics, their sensing behavior is generally dominated by optical pathways, and electronic readout often requires additional conductive supports or hybrid architectures. In contrast, MQDs inherit an intrinsically conductive transition-metal carbide framework together with quantum-confined and surface-state-mediated optical activity. This combination allows MQDs to sustain both carrier transport and emissive recombination within the same nanoscale platform.^35–39^ Moreover, the chemically diverse surface terminations of MQDs, including –O, –OH, –F, and heteroatom-derived functionalities, provide a broader and more electronically active interfacial landscape than that typically available in CDs. As a result, MQDs are not simply alternative fluorescent dots, but structurally integrated materials in which optical and electronic transduction can be co-designed at the atomic and surface levels, making them especially suitable for robust dual-mode sensing applications.

Table 1 summarizes the principal electronic and interfacial mechanisms governing the dual-mode sensing behavior of N-MQDs. The listed mechanisms collectively demonstrate that the sensing response is not controlled by a single electronic pathway, but rather by the synergistic interaction between band structure modulation, carrier transport, and surface-state engineering. Nitrogen incorporation modifies the local electronic configuration and redistributes charge density within the MXene lattice, thereby influencing the density of states near the Fermi level and enhancing charge-transfer efficiency. Simultaneously, quantum confinement and defect-induced trap states regulate carrier lifetime and recombination kinetics, which directly affect both optical emission and electrical conductivity. These interconnected processes establish the fundamental basis for simultaneous photonic and electronic signal generation in dual-mode sensing systems.

**Table 1:** Electronic–photonic coupling in N-MQDs

No.	Subsection	Core mechanism	Impact on sensing	Potential applications
1	Band structure modulation induced by nitrogen doping	Nitrogen atoms introduce localized electronic states within the MXene bandgap, modulating conduction and valence band edges	Coexistence of delocalized metallic conduction and localized radiative states enables simultaneous dual-mode optical/electronic responses	Multiplexed sensing platforms, optoelectronic devices, environmental monitoring
2	Charge carrier dynamics and competing radiative–nonradiative pathways	Dopant-induced trap states regulate electron–hole recombination, balancing radiative emission and nonradiative decay	Extended carrier lifetimes enhance photonic output while maintaining electrochemical accessibility, ensuring robust dual-mode signal generation	Real-time environmental sensing, bioelectronic transducers, point-of-care diagnostics
3	Density of states engineering and Fermi level realignment	Nitrogen incorporation modifies DOS near the Fermi level, shifting electronic occupation and excitation pathways	Improved overlap between conduction states and excited states enhances both optical emission and charge transfer efficiency	Ratiometric fluorescence sensors, multi-analyte detection, energy conversion devices
4	Coupling between localized dopant states and delocalized conduction networks	Hybridization between localized nitrogen orbitals and MXene conduction networks enables coherent electron transfer between emission and conduction pathways	Enables synchronous optical and electronic signaling, avoiding mutual interference of modalities	Multiplexed dual-mode sensing, hybrid photonic-electronic devices, integrated chemical sensors
5	Energetic decoupling as a prerequisite for signal orthogonality	Spatial and energetic separation of photonic and electronic states ensures semi-independent operation of both channels	Maintains signal orthogonality, allowing cross-validation without redundancy	High-fidelity environmental and biological sensing, multi-signal reporting, adaptive smart sensors
6	Surface functionalization and dopant positioning	Placement of nitrogen at edge *vs.* basal sites alters local electronic density and emission centers	Optimized functionalization improves signal intensity, stability, and selective analyte binding	Targeted chemical sensing, biosensing interfaces, tunable optoelectronic nanodevices
7	Quantum confinement effects on dual-mode emission	Size reduction induces discrete energy levels, amplifying sensitivity to dopant-induced electronic perturbations	Enhances both radiative efficiency and charge transfer probability, reinforcing dual-mode responses	Single-nanoparticle sensing, quantum dot optoelectronics, high-resolution imaging probes
8	Modulation of excited-state dynamics	Nitrogen dopants influence intersystem crossing and charge recombination rates	Allows fine-tuning of emission wavelength, intensity, and lifetime for optimized dual-mode readouts	Fluorescence lifetime-based sensors, time-resolved optical devices, dynamic analyte tracking
9	Multi-analyte signal encoding *via* state hybridization	Coupled localized-delocalized states permit multiplexed optical and electrochemical encoding	Enables detection of multiple analytes in a single probe while minimizing cross-talk	Multiplexed environmental sensors, wearable analytical platforms, high-throughput screening
10	Robustness against environmental perturbations	Broadening of DOS and decoupled channels provide graded response to pH, ionic strength, or temperature variations	Maintains stable dual-mode outputs under variable conditions	Field-deployable environmental monitoring, clinical diagnostic devices, robust sensing in complex matrices

Furthermore, the mechanisms presented in Table 1 indicate that interfacial coupling and energetic decoupling play critical roles in stabilizing sensing performance and improving signal reliability. Strong interfacial interactions between analytes and active N-MQD sites facilitate rapid electron exchange, while controlled energetic separation between radiative and nonradiative pathways minimizes signal interference and enhances sensitivity. The coexistence of conductive pathways and emissive centers enables complementary detection modes that improve selectivity, reproducibility, and detection accuracy under complex operating environments. Therefore, Table 1 provides an integrated mechanistic framework linking electronic structure engineering with multifunctional sensing performance, emphasizing that precise control of defects, heteroatom doping, and carrier dynamics is essential for the rational design of next-generation MXene-based dual-mode sensing platforms.

## 3. Structural and surface-state engineering for dual-mode signal integration

### 3.1. Defect landscape engineering as a design variable for dual-mode regulation

In N-MQDs, defects should not be interpreted merely as unavoidable structural imperfections, but rather as programmable elements that define the accessibility and balance of multiple sensing modes. From an engineering perspective, the defect landscape governs how charge carriers are temporally stored, redirected, or dissipated before contributing to measurable signals. Consequently, defect management becomes a central lever for enabling stable dual-mode behavior. The effectiveness of defect engineering lies in controlling defect distribution rather than minimizing defect density. Uniformly distributed defects can facilitate homogeneous carrier mediation across the quantum dot, whereas clustered defects often dominate recombination processes and bias the system toward a single transduction pathway.^46–48^ Additionally, defects with moderate energetic depth may serve as transient carrier buffers that synchronize optical and electronic responses without permanently trapping charges.

Disentangling the respective contributions of surface states, defect states, and nitrogen dopants in governing the dual-mode sensing behavior of N-MQDs remains experimentally challenging because these features are often intrinsically coupled during synthesis. Nevertheless, several studies have attempted to partially decouple these effects through systematic control strategies. A common approach involves comparing undoped MQDs with nitrogen-doped counterparts to evaluate the influence of heteroatom incorporation on electronic conductivity and photoluminescence characteristics. In addition, controlled variation of nitrogen precursor concentration during synthesis has been used to modulate doping levels, allowing correlations between nitrogen content and changes in carrier lifetime, emission intensity, or charge-transfer kinetics to be examined through techniques such as XPS, PL, and TRPL spectroscopy.^41–44^ Surface termination engineering, including modulation of –O, –OH, or –F functional groups, has also been explored to distinguish surface-state-dominated emission from defect-related recombination pathways. Although complete experimental isolation of these contributions remains difficult, these comparative and surface-engineering strategies provide valuable insight into the relative roles of defects, surface chemistry, and nitrogen dopants in regulating the coupled electronic and optical responses of N-MQD-based sensing platforms.

In addition to nitrogen-induced defect states, the surface terminations of MXene substrates (–O, –OH, –F) play a decisive role in governing analyte binding affinity and interfacial charge-transfer dynamics in hybrid N-MQD/MXene sensing systems. Oxygen- and hydroxyl-terminated surfaces generally enhance hydrophilicity, increase the density of Lewis-basic adsorption sites, and promote stronger hydrogen-bonding or dipole–dipole interactions with polar analytes. These terminations also facilitate more efficient charge extraction from N-MQDs by providing energetically favorable pathways for electron or hole transfer. In contrast, –F terminations exhibit more electron-withdrawing character, often reducing surface conductivity, suppressing carrier mobility, and weakening charge-transfer coupling. Importantly, these surface terminations interact synergistically with nitrogen dopants within MQDs.^46–49^ Nitrogen incorporation locally redistributes electronic density, alters the energy alignment between MQDs and MXene surfaces, and generates additional active sites that couple with O/OH groups to strengthen analyte adsorption and accelerate interfacial charge transfer. Conversely, in the presence of F terminations, the same nitrogen-induced states may experience partial charge localization that diminishes sensing sensitivity. Overall, the combined effects of MXene surface chemistry and nitrogen doping jointly regulate adsorption energies, local band structure, and charge-transfer kinetics, thereby enabling tunable and mode-dependent sensing performance in N-MQD/MXene hybrids.

In addition to the overall nitrogen content, the specific bonding configurations of nitrogen play a critical role in modulating the electronic structure and photoluminescence characteristics of N-doped MQDs. Spectroscopic analyses, particularly high-resolution X-ray photoelectron spectroscopy (XPS), typically reveal the coexistence of pyridinic, pyrrolic, and graphitic-like nitrogen species within the MQD framework. These configurations influence the electronic structure in distinct ways. Pyridinic nitrogen, located at edge or defect sites, introduces localized electronic states near the Fermi level that can act as charge trapping centers, thereby facilitating radiative recombination and contributing to enhanced photoluminescence intensity. Pyrrolic nitrogen is commonly associated with surface-related functional groups and can modify the local surface polarity, which affects the distribution of surface emissive states and may lead to shifts in emission wavelength. In contrast, graphitic-like nitrogen is incorporated into the basal lattice, where it improves electronic conductivity and promotes more efficient charge transport across the quantum dot structure. Although establishing precise quantitative correlations between individual nitrogen configurations and specific optical parameters remains challenging due to overlapping spectral contributions, several reports indicate that higher fractions of pyridinic and pyrrolic nitrogen are frequently associated with increased quantum yield and noticeable emission wavelength shifts. These observations suggest that controlled nitrogen configuration engineering provides an effective strategy for tuning both the electronic and luminescent properties of N-MQDs.

**Fig. 5:**
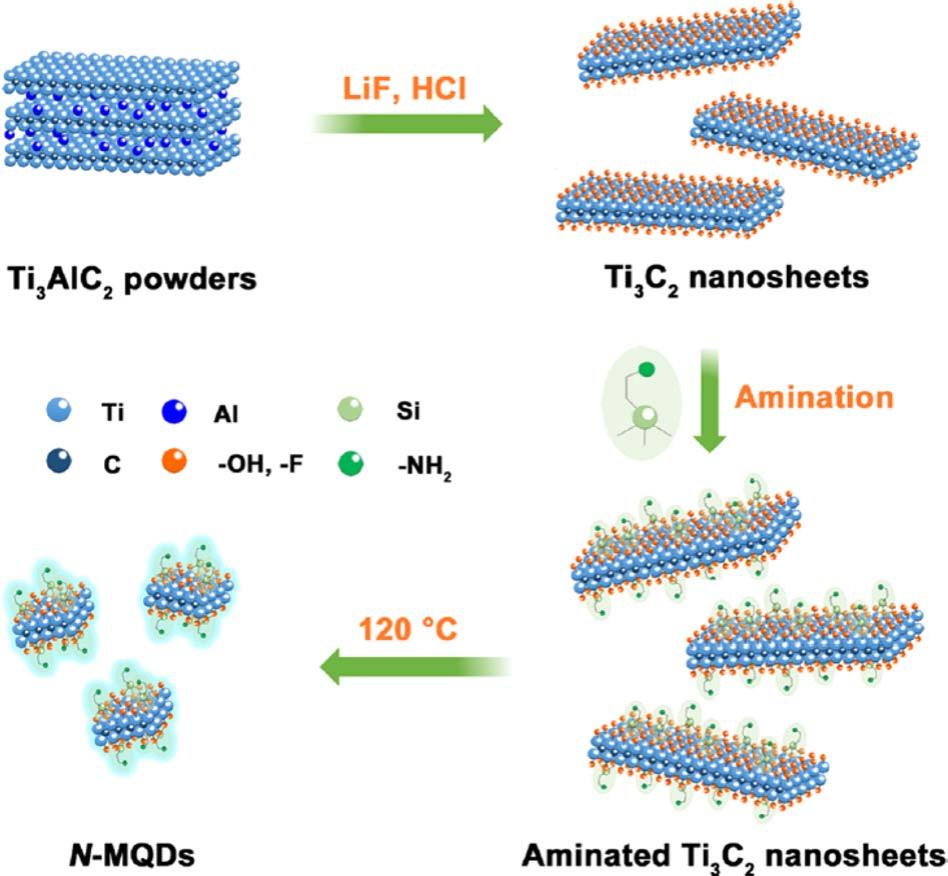
Synthetic route for nitrogen-doped Ti_3_C_2_T_*x*_ MXene quantum dots (N-MQDs) illustrating deliberate defect landscape engineering *via* APTES-assisted surface amination and hydrothermal cutting at 120 °C (pH 9). The process introduces controlled nitrogen-related defects (substitutional, surface –NH_2_, edge vacancies) as programmable elements to regulate carrier trapping, synchronization of radiative and nonradiative pathways, and ensemble uniformity for stable dual-mode transduction. Adapted with permission from ref. 49. © 2022 American Chemical Society.

Fig. 5 schematically illustrates the synthesis of N-Ti_3_C_2_T_*x*_ MQDs and emphasizes the role of the hydrothermal cutting process as an intentional defect-engineering strategy rather than a purely procedural step. Starting from etched and delaminated Ti_3_C_2_T_*x*_ nanosheets, APTES-derived –NH_2_ groups act as both nitrogen sources and surface modifiers prior to hydrothermal fragmentation at 120 °C under mildly basic conditions. This step inherently generates lattice distortions, under-coordinated sites, and nitrogen-related defects that modulate carrier trapping and transport. While the schematic highlights the conceptual link between synthesis and dual-mode functionality, it also idealizes the process; the exact nitrogen configuration and defect distribution remain unresolved and require complementary experimental validation to ensure reproducible sensing performance.

The incorporation of nitrogen atoms into MQDs fundamentally alters the local electronic structure and defect energetics, providing the physicochemical basis for the material effects discussed in this section. Nitrogen possesses higher electronegativity than carbon and introduces localized electronic perturbations when incorporated into the Ti_3_C_2_T_*x*_ lattice through substitutional, interstitial, or surface-bound configurations. These configurations generate heteroatom-induced states near the Fermi level and modify the density of states, which directly influences charge redistribution and carrier transport behavior. In substitutional configurations, nitrogen can replace carbon atoms in the carbide framework, leading to local lattice distortion and the formation of donor-like electronic states that enhance electron density around Ti centers. Surface-grafted nitrogen species such as –NH_2_ groups, on the other hand, primarily modify surface dipole moments and interfacial charge-transfer kinetics. Collectively, these nitrogen-derived electronic states act as tunable trap levels that regulate carrier capture, release, and recombination dynamics. This modulation of carrier pathways is particularly important in quantum-confined systems where small variations in electronic structure strongly influence optical emission and electronic conductivity. Consequently, nitrogen incorporation provides a controllable route for tailoring defect-mediated carrier dynamics, enabling the coordinated regulation of radiative and nonradiative processes required for stable dual-mode signal generation.

### 3.2. Surface termination programming for signal routing, selectivity, and robustness

Surface terminations define the immediate electronic and chemical interface through which signals are initiated, routed, and collected.^50,51^ In dual-mode MQD systems, surface chemistry serves as a programmable boundary layer that mediates how carriers engage with optical emission centers *versus* electronic extraction pathways. Importantly, surface terminations can influence mode preference without altering the intrinsic electronic structure of the quantum dot core. From a design perspective, surface termination programming enables selective amplification of one transduction route while preserving the viability of the other. Certain surface functionalities enhance interfacial charge transfer kinetics, facilitating electronic readout, while others passivate nonradiative traps and stabilize emissive excited states. Balancing these effects allows dual outputs to remain accessible without competitive suppression.^52–54^

Equally important is the role of surface terminations in mitigating environmental perturbations. As the primary interaction layer, surface chemistry buffers the quantum dot against chemical noise that could otherwise bias signal generation. Well-controlled surface terminations therefore contribute not only to signal efficiency but also to operational robustness. In this context, surface terminations should be viewed as signal routers rather than passive modifiers.^55–57^ Their deliberate selection and distribution allow designers to dictate how information flows through the dual-mode system, preserving mode independence while enabling coherent response generation.

**Fig. 6:**
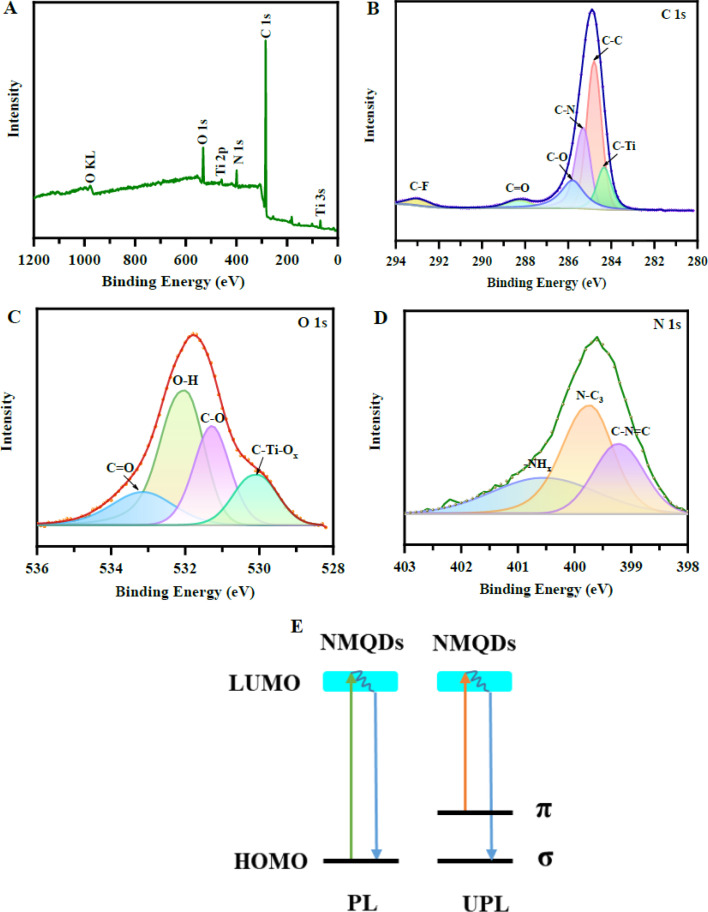
XPS characterization of nitrogen-doped Ti_3_C_2_T_*x*_ MXene quantum dots revealing a heterogeneous surface termination landscape for programmable signal routing and dual-mode selectivity. (A) Survey spectrum (Ti, C, O, N, F). (B) C 1s deconvolution (C–Ti, C–C/C=C, C–O, C–F, C–N). (C) O 1s species (O–H, C–O, C=O, C–Ti–O_*x*_). (D) N 1s spectrum showing pyridinic N–C_3_, pyrrolic C–N=C, and –NH_2_/–NH groups. (E) Schematic of down-conversion (π* → π) and up-conversion (σ → π*) PL mechanisms enabled by nitrogen-modified electronic states and anisotropic interfaces. Adapted with permission from ref. 53. © 2023 American Chemical Society.

Fig. 6 provides XPS evidence that the surface chemistry of N-Ti_3_C_2_T_*x*_ MQDs is chemically diverse and likely central to their dual-mode sensing behavior. The survey spectrum confirms the presence of Ti, C, O, N, and residual F, supporting the conclusion that nitrogen functionalization occurs on a surface already enriched with etching-derived terminations. This mixed termination environment is important because it can generate multiple adsorption sites, local polarity gradients, and distinct charge-transfer pathways, all of which may influence both electrochemical and photoluminescent responses.

The high-resolution C 1s and O 1s spectra further indicate coexistence of C–Ti, C–C/C=C, C–O, C=O, O–H, and Ti–O_*x*_-related species. Rather than being a simple sign of surface complexity, this suggests that the MQDs possess a heterogeneous interfacial structure in which different functional groups may either promote carrier extraction or passivate recombination centers. However, XPS alone cannot determine whether these groups are spatially uniform or whether some domains dominate the measured response. As a result, the relationship between surface composition and sensing reproducibility remains only partially resolved.

The N 1s spectrum is the most informative feature, with components assigned to pyridinic-like, pyrrolic-like, and amino-type nitrogen species. This distribution supports the argument that nitrogen is incorporated through more than one chemical pathway, which is mechanistically significant because different nitrogen configurations are expected to produce different electronic effects. Electron-rich nitrogen centers may facilitate interfacial charge transfer, whereas amino functionalities may contribute more strongly to surface passivation and analyte interaction. Still, these assignments remain indirect, and XPS deconvolution cannot unambiguously distinguish substitutional doping from surface grafting without complementary structural evidence.

Panel (e) proposes that this tailored termination landscape supports both down-conversion and up-conversion photoluminescence. While conceptually persuasive, the mechanism remains schematic unless correlated with time-resolved spectroscopy and quantitative emission analysis. Overall, Fig. 5 is valuable not because it merely confirms nitrogen doping, but because it reveals that controlled surface heterogeneity may be the key variable governing selectivity, signal balance, and operational stability in N-MQD-based dual-mode sensors.

### 3.3. Spatial dopant configuration control beyond global doping levels

While overall nitrogen content is often emphasized, dual-mode integration is far more sensitive to how dopants are spatially and chemically configured within the quantum dot framework. Distinct dopant configurations influence carrier localization and coupling strength in fundamentally different ways, leading to divergent signal behaviors even at identical doping levels. Uniform dopant dispersion promotes predictable carrier mediation across the quantum dot, whereas heterogeneous or edge-biased configurations can localize activity and induce mode dominance. From a structural engineering standpoint, controlling dopant configuration enables modulation of interaction strength between emissive sites and conductive pathways without altering material composition.^58,59^

### 3.4. Interface architecture for efficient dual-mode signal extraction

Dual-mode sensing platforms rely on hierarchical architectures in which quantum dots interact with electrodes, substrates, or host matrices. The interface between these components critically determines how signals are extracted, amplified, and stabilized. Poor interface design can lead to aggregation-induced quenching, uneven carrier extraction, or signal attenuation across modes. Effective interface architecture balances proximity and isolation. Quantum dots must be sufficiently connected to conductive supports to enable electronic readout, yet remain spatially separated enough to preserve individual emissive behavior. Controlled anchoring strategies prevent uncontrolled aggregation while maintaining electrical continuity.^60,61^

Interface homogeneity is equally important. Variations in contact geometry or coupling strength create spatially dependent signal bias, undermining dual-mode consistency. Rational interface design seeks uniform interaction conditions across the sensing ensemble, enabling synchronized optical and electronic responses. Rather than acting as passive scaffolds, interfaces function as active signal modulators. Their architecture defines the operational envelope of dual-mode platforms, translating intrinsic material capabilities into system-level performance.^62,63^

### 3.5. Crosstalk suppression through multidimensional design constraints in dual-mode systems

One effective strategy for suppressing mutual interference between sensing modes is spatial compartmentalization. By confining optical and electronic processes to partially distinct regions within the quantum dot or its surrounding architecture, competitive carrier consumption can be minimized. This spatial separation enhances mode independence without eliminating necessary coupling. Compartmentalization can occur at multiple length scales, from nanoscale segregation of emissive and conductive domains to mesoscale organization within composite matrices. The objective is not complete isolation but controlled interaction that preserves signal coherence while preventing dominance.^64–67^

Spatially compartmentalized designs also improve system robustness. Localized perturbations affect only specific signal pathways rather than collapsing overall functionality. This redundancy is particularly valuable in multimodal sensing environments subject to fluctuating conditions.^68–72^ By incorporating spatial design principles, dual-mode MXene QD systems can achieve higher signal fidelity and diagnostic reliability. Mode independence becomes an engineered feature rather than an incidental outcome.

### 3.6. Crosstalk suppression through multidimensional design constraints

Crosstalk suppression represents the culmination of structural and surface-state engineering efforts. Effective dual-mode systems must generate a correlated yet non-redundant signal, which requires careful alignment of energetic, spatial, and interfacial parameters. Single-parameter optimization is insufficient; instead, multidimensional constraints must be imposed to balance competing processes. Energetic alignment prevents one mode from monopolizing carrier populations, while spatial design limits direct competition. Interface architecture ensures consistent signal extraction, and defect management regulates carrier lifetimes. Together, these constraints define a multidimensional design space within which dual-mode behavior is stable.

Importantly, crosstalk suppression is not an afterthought but a guiding principle of system design. Dual-mode platforms that neglect this principle risk producing signals that are nominally distinct but functionally redundant.^73–76^ True dual-mode sensing emerges only when each signal contributes complementary information. By framing crosstalk suppression as a design objective rather than a problem to be mitigated, next-generation MXene QD platforms can achieve the robustness and clarity required for advanced multimodal sensing.

The Table 2 systematically summarizes the structural and surface-state engineering strategies that underpin dual-mode signal integration in N-MQDs. Each row identifies a distinct design principle—from defect landscape modulation and dopant configuration to interface architecture and surface reconstruction—clarifying how these nanoscale features control carrier behavior, emission pathways, and interfacial charge transfer. The mechanistic role column emphasizes the functional translation of atomic-scale modifications into macroscopic signal outcomes, highlighting how programmable defects, selective surface terminations, and spatial compartmentalization balance radiative and nonradiative processes. Crucially, these strategies collectively ensure dual-mode coherence: optical and electronic channels remain orthogonal yet correlated, allowing simultaneous multiplexed sensing without competitive suppression. The inclusion of dynamic features such as surface reconstruction and edge/basal plane functionalization illustrates that N-MQDs can adapt to environmental perturbations, providing robustness in complex matrices. This table not only organizes the current state-of-the-art mechanistic understanding but also offers a rational design roadmap for next-generation multimodal MQDs, linking structural engineering principles directly to dual-mode sensing performance, selectivity, and operational fidelity. By integrating defect, dopant, interface, and spatial considerations, it provides a comprehensive framework for translating nanoscale modifications into predictable, high-performance sensing outcomes suitable for environmental, biomedical, and analytical applications.

**Table 2:** Structural and surface-state engineering strategies for dual-mode signal integration in N-MQDs

Focus	Structural/Surface feature	Mechanistic role	Dual-mode relevance
Defect landscape engineering	Programmable vacancies and edge defects	Modulate carrier storage, redistribution, and recombination	Balances optical and electronic responses, stabilizes dual-mode output
Surface termination programming	Functional groups (–OH, –F, –O, amines)	Route carriers selectively, passivate nonradiative traps	Enhances mode independence and environmental robustness
Dopant configuration control	Spatially localized N atoms	Tune carrier localization, modulate emissive-conductive coupling	Maintains dual-mode coexistence, prevents mode collapse
Interface architecture	Electrode/host matrix anchoring	Control signal extraction and amplification, prevent aggregation	Ensures synchronous signal generation, improves reproducibility
Spatial compartmentalization	Nanoscale segregation of emissive *vs.* conductive domains	Reduces competitive carrier consumption	Preserves signal orthogonality and mode fidelity
Crosstalk suppression	Multidimensional energetic, spatial, interfacial alignment	Minimize mode interference	Guarantees correlated yet non-redundant dual signals
Surface strain and lattice distortion	Localized lattice perturbations	Modify electronic states, facilitate selective trapping	Enhances selective excitation for dual outputs
Edge *vs.* basal plane functionalization	Differential functional group placement	Direct carrier flow toward specific transduction channels	Provides spatial routing for multimodal signal integration
Dynamic surface reconstruction	Responsive surface groups under stimuli	Enable adaptive emission and charge transfer	Supports real-time dual-mode sensing under variable conditions

## 4. Dual-mode and multiplexed sensing applications enabled by N-doped MXene quantum dots

### 4.1. Reversible coordination-driven on/off/on fluorescence encoding for arsenic detection

Nitrogen-doped Ti_3_C_2_ MXene quantum dots (N-MQDs) exhibit a distinctive “on/off/on” fluorescence response, making them an attractive dual-mode platform for arsenic monitoring. The bright yellow emission at 570 nm provides superior signal-to-noise ratio and deeper penetration compared to conventional blue-emitting MQDs, which is particularly advantageous for environmental and biological matrices where interference from background autofluorescence is significant. Upon addition of As^3+^ ions, the fluorescence is quenched due to static quenching, which occurs *via* complex formation between the surface functional groups of N-MQDs and the arsenic ions. This process is highly selective, fast, and reproducible, establishing the basis for precise analyte recognition.^77^

Fluorescence recovery is initiated by 2-amino-6-methoxybenzothiazole (MBTZ), which binds preferentially to As^3+^, effectively displacing it from the N-MQDs surface and restoring fluorescence. This reversible “on/off/on” mechanism encodes dual information within a single nanosensor, enabling simultaneous qualitative and quantitative detection. The platform demonstrates low detection limits of 30 nM for As^3+^ and 0.44 µM for MBTZ, highlighting its sensitivity and suitability for trace-level analysis in complex aqueous environments. Additionally, the system maintains its performance across a wide pH range and in matrices containing interfering ions, suggesting high robustness for practical applications.

Integration into solid-state formats, such as thin films or paper-based devices, enhances portability and on-site applicability. The real-time fluorescence modulation allows for continuous monitoring without the need for complex instrumentation or repeated reagent addition, which is advantageous for field deployment. Mechanistic studies reveal that the dynamic and static quenching contributions, combined with the reversible complexation with MBTZ, enable precise control over signal modulation. Overall, this study underscores the potential of N-MQDs as multifunctional, reversible probes for environmental sensing, capable of rapid, selective, and sensitive arsenic detection while minimizing reagent consumption and operational complexity.

### 4.2. Paper-based adsorption and sensing of dichromate ions

Nitrogen and boron co-doped MXene quantum dots (NB-MQDs) immobilized on polyethyleneimine-functionalized pulp fiber paper (NB-MQDs@PP) provide an innovative dual-function platform that merges adsorption and sensing of Cr_2_O_7_^2−^. NB-MQDs demonstrate improved oxidation stability and extended excitation wavelengths compared to undoped MQDs, facilitating rapid optical response and reduced photobleaching. The paper substrate not only provides a mechanically robust and flexible support but also contributes functional groups to enhance interaction with Cr_2_O_7_^2−^ ions.^78^

The sensing performance is remarkable, with response times of 10 seconds and quenching efficiencies reaching 99.9%. Two distinct detection strategies, immersion and cyclic filtration, enable quantitative analysis, with detection limits of 17.0 nM and 3.8 nM, respectively. These figures surpass most reported approaches for dichromate detection, reflecting the high sensitivity imparted by the NB-MQDs. Simultaneously, the platform demonstrates impressive adsorption capacity of 162.4 mg g^−1^, highlighting its environmental remediation potential. The dual-mode behavior—adsorbing and signaling the analyte—allows the device to act as both a capture medium and a real-time monitor, a critical advantage in decentralized water quality assessment. Mechanistic insights indicate that the doping of nitrogen and boron enhances charge transfer interactions with Cr_2_O_7_^2−^ and stabilizes the photoluminescence, enabling efficient quenching and rapid signal output. The modular design of NB-MQDs@PP also allows customization for specific field applications, such as varying thickness or functionalization to accommodate different contaminants. This study demonstrates a new paradigm in MXene-based nanomaterials, where smart paper substrates not only detect toxic ions with high sensitivity but simultaneously remove them from solution, merging environmental sensing with remediation in a single, practical platform.

### 4.3. Ratiometric FRET-based detection of copper and d-penicillamine

Ratiometric sensing using N-MQDs leverages fluorescence resonance energy transfer (FRET) to simultaneously detect copper ions (Cu^2+^) and d-penicillamine (D-PA). In this system, N-MQDs act as energy donors and the oxidation product 2,3-diaminophenazine (ox-OPD) serves as an acceptor. Upon oxidation of *o*-phenylenediamine by Cu^2+^, ox-OPD is generated, which quenches N-MQDs emission at 450 nm while emitting at 570 nm. The resulting inverse FRET signal enables ratiometric analysis, improving reliability and minimizing artifacts from environmental fluctuations or probe concentration variations.^79^ The addition of D-PA inhibits the Cu^2+^-mediated oxidation, thereby modulating the ratio of donor and acceptor fluorescence. This reversible dual-mode mechanism provides high selectivity for both Cu^2+^ and D-PA, with detection limits of 3.0 nM and 0.115 µM, respectively. By exploiting the ratiometric FRET principle, the sensor achieves both high sensitivity and robustness, making it suitable for complex chemical matrices where multiple species might interfere.

This system underscores the potential of N-MQDs as versatile energy donors capable of precise ratiometric control. The dual-analyte detection is particularly relevant for biomedical and environmental applications, where simultaneous monitoring of metal ions and biologically active molecules is critical. Additionally, the platform's photostability and long-wavelength emission enhance practical applicability, allowing for real-time detection without significant photobleaching. The study demonstrates how molecular design, combined with N-MQDs’ unique optical properties, can produce dual-mode nanosensors with analytical precision and operational simplicity.

### 4.4. Fluorescence nanoprobes for simultaneous Co^2+^ and Ag^+^ sensing

N-Ti_3_C_2_ MQDs, synthesized *via* inorganic base stripping, function as dual-analyte fluorescent nanoprobes for cobalt (Co^2+^) and silver (Ag^+^) ions, capitalizing on their tunable photoluminescence and surface functionalization. Upon exposure to either ion, the emission intensity of N-MQDs is substantially quenched due to combined effects of static quenching and inner filter absorption. Static quenching arises from coordination between Co^2+^/Ag^+^ ions and the amino or hydroxyl functional groups on the N-MQDs surface, leading to formation of non-emissive complexes. Concurrently, the inner filter effect further attenuates the fluorescence by absorption of emitted photons by the metal ions.^80^ These dual mechanisms enable precise, simultaneous quantification of both metal ions in aqueous media with detection limits of 0.21 µM (Co^2+^) and 0.10 µM (Ag^+^).

The probe exhibits remarkable photostability and retains bright blue emission, even under prolonged excitation, facilitating deployment in real-world water samples without significant signal decay. Mechanistic studies reveal that quenching efficiency depends on the specific coordination environment and electronic interactions between the doped MXene lattice and the analytes, highlighting the importance of controlled doping for maximizing sensitivity. The dual-analyte capability reduces analytical workload by consolidating multiple measurements into a single assay, streamlining environmental monitoring and industrial effluent analysis.

Furthermore, the facile and reproducible synthesis enables scalable production, ensuring that the platform can be applied for high-throughput monitoring. Integration into portable or solid-state devices could allow on-site detection, providing both environmental surveillance and early warning for metal contamination. Overall, this study illustrates how multifunctional N-MQDs combine tunable optical properties with selective binding, producing versatile, dual-mode fluorescent probes for real-time, multiplexed sensing applications.

### 4.5. Inner filter effect-based detection of Cr(vi) and ascorbic acid

Nitrogen-doped Ti_3_C_2_ MQDs exploit the inner filter effect to achieve sequential, dual-mode detection of chromium(vi) and ascorbic acid (AA), exemplifying temporal control of fluorescence signals. Cr(vi) ions strongly absorb the excitation or emission photons of N-MQDs, leading to quenching (“off” state), whereas AA reduces Cr(vi) to Cr(iii), restoring emission (“on” state). This reversible fluorescence modulation allows a single nanoprobe to report two chemically distinct analytes in a temporal sequence, enhancing analytical efficiency.^81^

The quenching involves both static interactions and photon absorption, with surface functional groups of N-MQDs forming complexes with Cr(vi). Fluorescence recovery occurs when AA reduces Cr(vi), breaking the complex and releasing the probe. Detection limits are exceptionally low—0.012 µM for Cr(vi) and 0.02 µM for AA—with a wide linear range (0.1–500 µM), demonstrating both sensitivity and versatility. The probe's stability under varying pH and ionic strength ensures robustness in complex environmental samples.

Mechanistic analysis emphasizes the strategic exploitation of N-MQDs’ optical properties and functionalized surfaces to encode dual information in a single system. This approach minimizes reagent consumption and reduces experimental complexity. Its practical application is evident in environmental monitoring of redox-active pollutants, where sequential detection of oxidants and reductants is critical. By integrating signal reversibility with high photostability, this platform offers a dynamic and reliable sensing strategy, suitable for environmental and food safety surveillance, as well as potential clinical diagnostic applications.

**Fig. 7:**
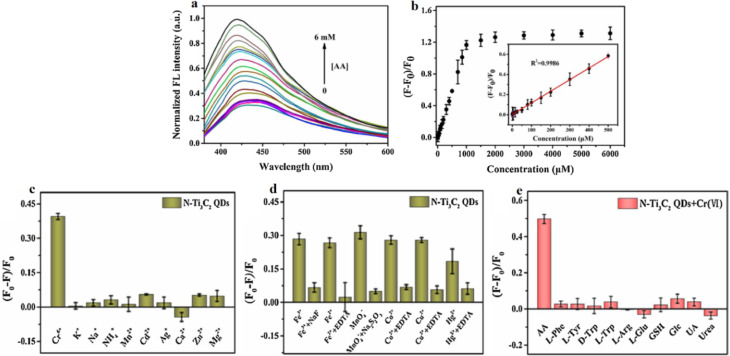
Performance of the N-Ti_3_C_2_ MQDs-Cr(vi) nanoprobe for sensitive and selective ascorbic acid (AA) detection *via* fluorescence recovery (inner filter effect reversal). (a) Normalized emission spectra showing progressive recovery at ∼450 nm with increasing AA concentration (0–6.0 mM). (b) Fluorescence recovery efficiency ((*F* − *F*_0_)/*F*_0_) *vs.* AA concentration; inset: linear calibration (*R*^2^ = 0.9986, 0.1–500 µM, LOD = 0.02 µM). (c) and (d) Selectivity of Cr(vi) quenching against interferents before/after masking with chelators (EDTA, NaF, Na_2_S_2_O_3_). (e) AA detection selectivity against amino acids, biomolecules, and reductants (2.0 mM, GSH 0.5 mM). The “on–off–on” behavior confirms redox-mediated Cr(vi) reduction and restoration of N-MQDs photoluminescence. Adapted with permission from ref. 81. © 2021 Elsevier B.V.

Fig. 7 evaluates the analytical performance of the N-Ti_3_C_2_ MQDs–Cr(vi) nanoprobe for IFE-based “on–off–on” fluorescence modulation and sequential sensing of Cr(vi) and ascorbic acid (AA). Its main contribution is not the visual recovery of emission *per se*, but the performance evidence that quenching and recovery can be made sufficiently strong, selective, and reversible to serve as a practical two-analyte workflow.

In panel (a), the gradual restoration of the ∼450 nm emission with increasing AA is consistent with removal of the IFE absorber (Cr(vi)) *via* reduction to Cr(iii). The interpretation is plausible, but it remains partially inferential: IFE dominance should be validated by directly showing excitation/emission overlap with Cr(vi) absorption and demonstrating that fluorescence lifetime is largely unchanged upon Cr(vi) addition (IFE/static absorption) compared with dynamic quenching. Without lifetime or corrected absorbance controls, residual contributions from surface complexation, redox-induced surface chemistry changes, or aggregation cannot be excluded, particularly given the heteroatom-rich surface evidenced earlier.

Panel (b) provides the quantitative merit figures for AA recovery (linear range, *R*^2^ and LOD). While the reported linearity and low LOD are attractive, two limitations are implicit in the curve shape: (i) the response saturates beyond ∼ mM levels, suggesting a finite Cr(vi) “reservoir” and constraining quantification at higher AA; and (ii) the calibration depends on the initial quenched state, so batch-to-batch reproducibility and timing (how long after quenching AA is introduced) become critical variables. Reporting intra/inter-day precision, probe stability, and recovery kinetics would strengthen claims of operational robustness.

Panels (c) and (d) address selectivity for Cr(vi) by surveying interferents and introducing masking agents. This is a strong practical step, but it also signals that selectivity is conditional: the assay's discrimination relies partly on added chelators (EDTA, NaF, *etc.*). In real matrices, unknown ligands and variable ionic strength can alter both Cr(vi) speciation and surface binding; thus, the apparent “exclusive” response may shift. Moreover, masking agents may themselves affect MQD surface states or optical background, so controls showing negligible impact on baseline fluorescence are necessary. Panel (e) suggests high specificity of AA in restoring fluorescence. However, the test set includes only limited reductants and uses unequal concentrations (*e.g.*, GSH), making it difficult to generalize selectivity across clinically or environmentally relevant reducing agents. Comparative redox potential/kinetics discussion and experiments in real samples (spiked recovery) would better substantiate the claimed uniqueness.

### 4.6. Sequential coordination and chelation-regulated off–on–off fluorescence sensing of Zn^2+^ and oxalic acid

Ultrasonically synthesized N-MQDs exhibit a reversible “off–on–off” fluorescence behavior, enabling sequential detection of Zn^2+^ ions and oxalic acid (OA). Fluorescence activation (“on”) occurs *via* coordination of Zn^2+^ with surface sites on N-MQDs, enhancing intramolecular charge transfer and stabilizing the excited state. Subsequent addition of OA quenches the fluorescence (“off”) through formation of a stable Zn^2+^–oxalate complex, which perturbs electron transfer and inhibits emission. This dual modulation allows precise temporal encoding of two analytes using a single nanoprobe.^82^

The system demonstrates rapid response kinetics and high selectivity, with detection limits of 0.127 µM for Zn^2+^ and 0.883 µM for OA. Mechanistic studies highlight the critical role of competitive binding: the strong Zn^2+^–OA interaction disrupts the previously stabilized fluorescent state, providing reversible and reproducible signal switching. Such a design exploits the tunable coordination chemistry of MQDs, enabling multiplexed sensing without requiring multiple probes or additional reagents. This “off–on–off” approach exemplifies the versatility of N-MQDs in complex analytical environments. It combines tunable photoluminescence, selective metal ion recognition, and dynamic signal encoding to facilitate real-time monitoring of trace metals and small organic molecules. The platform's simplicity, rapidity, and adaptability make it highly relevant for environmental monitoring, food safety, and potential clinical diagnostics, offering both operational efficiency and analytical depth.

To strengthen the mechanistic interpretation of dual-mode sensing systems, a quantitative comparison of structure–property relationships was performed (Table 3). The collected data reveal a direct correlation between heteroatom doping, photophysical modulation, and sensing performance in N-doped MXene quantum dots (N-MQDs). Nitrogen doping consistently enhanced fluorescence quantum yield (13.8–14.46%), photostability, and visible-range emission, while co-doping with boron further improved oxidation resistance and excitation wavelength extension. These electronic and photonic modifications strongly influenced carrier-transfer dynamics and sensing sensitivity. For example, FRET-based systems exhibited ultralow detection limits down to 3.0 nM owing to efficient donor–acceptor energy transfer enabled by spectral overlap, whereas inner filter effect (IFE)-dominated systems achieved reversible fluorescence modulation with detection limits as low as 0.012 µM. Similarly, coordination-induced intramolecular charge transfer (ICT) in Zn^2+^ sensing produced selective fluorescence enhancement, while competitive chelation suppressed carrier transfer and emission recovery. Quantitative comparison further demonstrates that co-doped and surface-engineered MQDs provide faster response kinetics, stronger quenching efficiencies, and improved analyte enrichment capacities, confirming that sensing performance is fundamentally governed by doping-regulated electronic structure and interfacial charge-transfer mechanisms.

**Table 3:** Quantitative structure–property relationships and mechanism–performance correlations of N-MQDs in dual-mode and multiplexed sensing systems

MQD structure/doping strategy	Key optical/electronic properties	Dominant sensing mechanism	Target analyte(s)	Quantitative analytical performance	Structure–property relationship	Ref.
Hydrothermally synthesized N-doped Ti_3_C_2_ MQDs	Yellow emission at 570 nm; excitation at 420 nm; QY = 13.8%	Static quenching + reversible coordination recovery	As^3+^/MBTZ	LOD = 30 nM (As^3+^), 0.44 µM (MBTZ)	N-Doping induced red-shifted emission and enhanced visible photoluminescence, while surface functional groups enabled reversible coordination-driven fluorescence modulation	77
N,B co-doped MQDs integrated into PEI-functionalized paper (NB-MQDs@PP)	Extended excitation wavelength; enhanced oxidation stability; quenching efficiency = 99.9%	Charge-transfer-assisted fluorescence quenching and adsorption	Cr_2_O_7_^2-^	Response time = 10 s; LOD = 1.2 µM (solution), 17.0 nM (immersion), 3.8 nM (cyclic filtration); adsorption capacity = 162.4 mg g^−1^	N/B co-doping improved electronic stability and optical response, while PEI-paper integration increased analyte enrichment and ultratrace detection sensitivity	78
Hydrothermal N-doped Ti_3_C_2_ MQDs	Donor emission = 450 nm; acceptor emission = 570 nm; high photostability	FRET-based ratiometric sensing	Cu^2+^/D-PA	LOD = 3.0 nM (Cu^2+^), 0.115 µM (D-PA)	Spectral overlap between donor N-MQDs and ox-OPD enabled efficient energy transfer and highly sensitive ratiometric fluorescence output	79
N-Doped Ti_3_C_2_ QDs prepared by KOH stripping	Particle size = 2.3 nm; QY = 14.46%; stable blue emission	Inner filter effect + static quenching	Co^2+^/Ag^+^	LOD = 0.21 µM (Co^2+^), 0.10 µM (Ag^+^)	Reduced particle size and N-doping enhanced fluorescence efficiency and promoted dual-ion-induced non-radiative quenching pathways	80
N-Doped Ti_3_C_2_ MQDs	Excitation-dependent photoluminescence; high photobleaching resistance	Inner filter effect + static quenching + redox recovery	Cr(vi)/AA	Linear range = 0.1–500 µM; LOD = 0.012 µM (Cr(vi)), 0.02 µM (AA)	Nitrogen doping stabilized emissive states and enabled reversible “on–off–on” fluorescence modulation through redox-mediated recovery	81
Ultrasonically synthesized N-MQDs	Particle size <10 nm; high water dispersibility; enhanced PL	ICT-enhanced fluorescence and competitive coordination quenching	Zn^2+^/OA	Linear range = 0–20 µM; LOD = 0.127 µM (Zn^2+^), 0.883 µM (OA)	Zn^2+^ coordination promoted intramolecular charge transfer (ICT), whereas Zn^2+^–oxalate chelation suppressed carrier transfer and fluorescence emission	82

### 4.7. Mechanistic insights and surface engineering of N-MQDs for multiplexed dual-mode sensing

N-MQDs have emerged as versatile nanomaterials, exhibiting tunable photoluminescence, high chemical stability, and excellent water dispersibility, which make them ideal for dual-mode and multiplexed sensing applications. The unique optical properties of N-MQDs arise primarily from the synergistic effects of nitrogen doping and the abundant surface functional groups, including hydroxyl, amine, and oxygen moieties. These functional groups not only modulate the electronic structure of the MXene core, enhancing visible-range emission, but also provide specific coordination sites for analytes such as heavy metals, metalloids, and small organic molecules.^78,79^

The “on/off/on” and “off–on–off” fluorescence behaviors observed in N-MQDs are largely governed by static quenching, inner filter effects (IFE), and fluorescence resonance energy transfer (FRET). For example, in arsenic sensing, static quenching occurs through the formation of non-emissive complexes between As^3+^ ions and surface functional groups, while fluorescence recovery is induced by competitive binding with MBTZ, highlighting the dynamic reversibility of surface–analyte interactions.^77^ Similarly, the inner filter effect, demonstrated in sequential Cr(vi) and ascorbic acid detection, illustrates how selective absorption of excitation or emission photons by analytes modulates the observed fluorescence intensity, allowing temporally resolved dual-analyte monitoring.^81^ In ratiometric FRET platforms for Cu^2+^ and d-penicillamine, the engineered spectral overlap between donor N-MQDs and acceptor ox-OPD facilitates precise ratio-metric readouts, enhancing detection fidelity and minimizing environmental interference.^79^

Surface engineering strategies play a pivotal role in achieving multiplexed sensing capabilities. Co-doping with boron, for instance, extends the emission wavelength of MQDs and enhances oxidation stability, enabling rapid and sensitive dichromate ion detection on smart paper-based substrates (NB-MQDs@PP) with dual adsorption-sensing functionality.^78^ Likewise, tuning particle size, doping concentration, and surface passivation modulates photoluminescence quantum yields, response times, and analyte specificity, as demonstrated in simultaneous Co^2+^/Ag^+^ detection, where both inner filter and static quenching mechanisms are leveraged for dual-target analysis in complex matrices.^80^ Ultrasonically synthesized N-MQDs exhibit reversible “off–on–off” behavior for Zn^2+^ and oxalic acid detection, where coordination-induced fluorescence activation and competitive binding govern the sequential signal modulation.^82^ These examples underscore the importance of rational surface functionalization and the integration of physicochemical insights into the design of highly selective, multi-analyte sensors.

Environmental conditions such as pH, ionic strength, and oxidative stability can significantly influence the sensing behavior and reliability of N-MQD-based dual-mode platforms. Variations in pH alter the protonation and deprotonation states of surface functional groups, including hydroxyl, amine, and oxygen-containing moieties on the MQDs and MXene lattice. These changes modify surface charge distribution, analyte binding affinity, and interfacial charge-transfer efficiency, thereby affecting both fluorescence intensity and electrochemical or electronic responses. Ionic strength also plays a critical role by influencing electrostatic screening and the structure of the electrical double layer at the sensing interface. Increased ionic concentrations may partially suppress analyte–surface interactions or alter carrier transport pathways, which can lead to variations in signal magnitude and detection sensitivity. In addition, the oxidative stability of MXene-based materials is an important factor governing long-term performance. Gradual oxidation may modify the electronic structure, introduce additional defect states, and reduce electrical conductivity, potentially leading to signal drift during extended operation.^78–80^ Nevertheless, several studies report that N-MQD hybrid systems retain stable optical responses across moderate pH ranges and in the presence of competing ions. The coexistence of optical and electrical detection modes further enhances reproducibility by enabling cross-validation of signals, thereby improving the overall robustness of dual-mode sensing platforms under variable environmental conditions.

From a mechanistic standpoint, the interactions between N-MQDs and analytes can be categorized into three complementary modes: (i) coordinate covalent interactions between surface nitrogen/oxygen groups and metal ions or metalloids, enabling selective binding; (ii) electron/energy transfer mechanisms, including FRET or photoinduced electron transfer, allowing ratiometric signal encoding; and (iii) dynamic modulation *via* competitive binding or redox reactions, which facilitates reversible “on/off/on” or sequential “off–on–off” fluorescence responses.^77,78^ These mechanisms not only underpin analytical sensitivity but also enable the simultaneous quantification of multiple analytes within a single assay, reducing sample handling complexity and reagent consumption.

In addition to photophysical considerations, the practical applicability of N-MQDs is enhanced through structural and economic design. Solid-state platforms, such as paper-based NB-MQDs@PP sensors, combine rapid detection, high adsorption capacity, and portability, aligning with on-site environmental monitoring needs.^78^ The facile synthesis methods, including hydrothermal, ultrasonication, and inorganic base stripping, allow scalable production while maintaining reproducibility and high quantum yields.^80,81^ Consequently, surface-engineered N-MQDs represent a synergistic integration of mechanistic understanding, structural tuning, and operational feasibility, providing an advanced framework for next-generation dual-mode, multiplexed sensing devices with applications ranging from environmental monitoring to food safety and clinical diagnostics.

The mechanistic insights reveal that surface chemistry and doping strategies are central to controlling dual-mode signal encoding, selectivity, and reversibility in N-MQDs. Rational engineering of these nanostructures enables the design of multifunctional sensors capable of simultaneous, sequential, and ratiometric detection, offering robust, high-performance platforms that meet the stringent analytical and practical demands of contemporary chemical sensing.

**Fig. 8:**
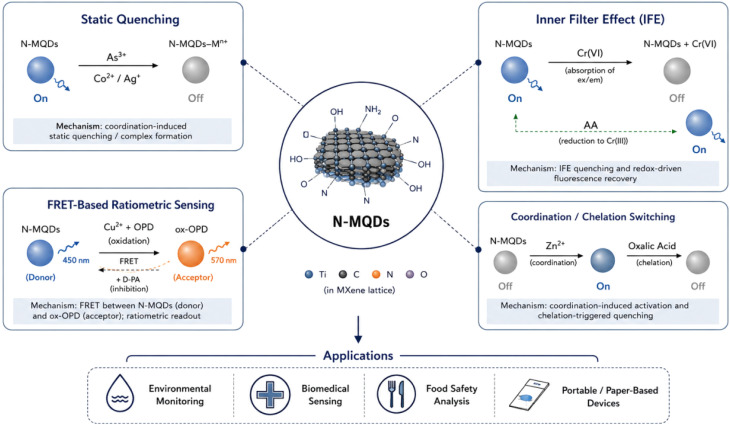
Schematic summary of dual-mode and multiplexed sensing strategies enabled by N-MQDs. Representative fluorescence modulation mechanisms, including static quenching, inner filter effect, FRET, and coordination/chelation switching, facilitate selective and reversible detection for environmental, biomedical, and portable sensing applications.

Fig. 8 summarizes the principal sensing mechanisms and multifunctional applications of N-MQDs. Owing to their tunable electronic structure, surface functional groups, and enhanced photoluminescence, N-MQDs enable diverse fluorescence modulation pathways including static quenching, inner filter effect (IFE), fluorescence resonance energy transfer (FRET), and coordination/chelation-regulated switching. These mechanisms support reversible, sequential, and ratiometric detection of multiple analytes within a single nanosensing platform. The schematic also highlights representative application areas such as environmental monitoring, biomedical sensing, food safety analysis, and portable paper-based sensing systems, illustrating the versatility and practical potential of N-MQD-based dual-mode sensing technologies.

### 4.8. Quantitative comparison with state-of-the-art dual-mode sensing platforms

To better contextualize the performance of N-MQD-based sensing systems, it is useful to compare their analytical characteristics with those of other recently reported dual-mode sensing platforms, including systems based on metal nanoparticles, metal–organic frameworks (MOFs), carbon quantum dots, and graphene-derived nanomaterials. Across the literature, many of these platforms demonstrate high analytical sensitivity and low detection limits due to their large surface area, tunable surface chemistry, and efficient charge-transfer properties. However, N-MQDs offer several distinctive advantages that make them particularly suitable for multiplexed dual-mode sensing applications.

In terms of sensitivity and detection limits, N-MQD-based sensors typically achieve detection limits in the nanomolar to micromolar range depending on the analyte and transduction mechanism, which is comparable to those reported for other carbon-based nanomaterials and hybrid nanostructures. The presence of nitrogen dopants introduces additional electronic states and surface defects that enhance fluorescence modulation and facilitate interfacial charge transfer, thereby improving signal responsiveness in both optical and electrochemical detection modes.^78–81^ Response times are generally rapid, often within seconds to a few minutes, owing to the small size of the quantum dots and the high accessibility of surface functional groups for analyte interaction.

Selectivity in N-MQD-based systems is primarily governed by surface functionalization strategies and heteroatom-induced electronic modulation. Compared with several conventional nanomaterial platforms, the tunable surface chemistry of N-MQDs enables more flexible integration of recognition elements or specific binding sites. Moreover, the coexistence of optical and electrical signal outputs provides an inherent advantage for multiplexed detection and cross-validation of sensing signals. This dual-readout capability can reduce false positives and improve measurement reliability, particularly in complex matrices. Overall, while the absolute analytical performance of N-MQD sensors may be comparable to that of other advanced nanomaterial-based sensing platforms, their key advantage lies in the synergistic integration of tunable surface chemistry, defect-engineered electronic structure, and intrinsically compatible dual-mode signal generation, which collectively support reliable multiplexed sensing in diverse analytical environments.

### 4.9. Scientific context and emerging role of N-MQD-based dual-mode sensors

Recent years have witnessed rapid growth in the development of dual-mode sensing platforms based on a variety of nanomaterials, including metal nanoparticles, metal–organic frameworks (MOFs), graphene derivatives, and carbon quantum dots. Several recent reviews have summarized these systems, emphasizing improvements in sensitivity, signal amplification strategies, and integration of optical and electrochemical transduction mechanisms. While these studies provide valuable insights into dual-mode sensing technologies, most focus broadly on general nanomaterial platforms and typically treat MXene-derived quantum dots only briefly or as part of larger material categories. Consequently, the unique contributions of nitrogen-doped MXene quantum dots (N-MQDs) to multiplexed sensing architectures have not yet been systematically discussed.

The present review addresses this gap by specifically focusing on N-MQDs as a distinct class of functional nanomaterials capable of enabling reversible, ratiometric, and sequential sensing mechanisms within a single nanoscale platform. Unlike many previously reviewed systems that rely on combining separate nanomaterials to achieve dual-mode detection, N-MQDs integrate multiple signal-modulation pathways—such as static quenching, inner filter effects, and FRET—within a single engineered nanostructure.^75–78^ This intrinsic multifunctionality simplifies sensor architecture while maintaining high analytical performance.

Another distinguishing aspect highlighted in this work is the role of nitrogen doping and surface engineering in tailoring the electronic structure and coordination chemistry of MXene quantum dots. These modifications introduce tunable defect states and abundant functional groups that facilitate selective analyte binding and controllable fluorescence modulation. As illustrated in the sensing strategies discussed in Sections 4.1–4.7, these properties enable diverse signal-encoding mechanisms including “on/off/on,” “off–on–off,” ratiometric, and sequential detection modes, which are particularly advantageous for multiplexed analysis.

By consolidating recent experimental studies and mechanistic insights, this review provides a focused framework that clarifies how N-MQDs bridge the gap between conventional quantum dot sensors and emerging multifunctional nanoplatforms.^79–81^ This perspective highlights their potential to advance the design of compact, reliable, and multiplexed dual-mode sensing systems for environmental, biomedical, and food safety applications.

## 5. Conclusion and future perspectives: towards next-generation dual-mode N-MQDs sensors

The recent advancements in N-MQDs have unequivocally demonstrated their immense potential as versatile platforms for dual-mode and multiplexed sensing. Through careful design of doping levels, surface functionalization, and structural engineering, N-MQDs can encode complex chemical information *via* fluorescence modulation, enabling sequential, reversible, and ratiometric detection of diverse analytes, including metal ions, metalloids, and small organic molecules.^32,78^ The body of work reviewed herein underscores the unique intersection of materials chemistry, photophysics, and analytical science, highlighting the multifaceted capabilities of these nanostructures in environmental monitoring, food safety, and biomedical diagnostics.

One of the most significant achievements of N-MQDs is their ability to perform multiplexed dual-mode sensing within a single platform. As evidenced by the “on/off/on” fluorescence response for arsenic and MBTZ detection,^77^ the ratiometric FRET-based detection of Cu^2+^ and d-penicillamine,^79^ and the “off–on–off” sequential detection of Zn^2+^ and oxalic acid,^82^ N-MQDs can simultaneously or sequentially monitor multiple analytes with high selectivity and low detection limits. These capabilities are primarily attributed to three interrelated factors: (i) the tunable electronic and photophysical properties induced by nitrogen (and co-doping),^78^ (ii) the abundance of surface functional groups providing specific binding sites for target analytes, and (iii) the dynamic interplay between quenching, energy transfer, and inner filter effects.^80,81^ Together, these mechanisms allow the nanomaterials to encode and decode chemical signals in highly controlled manners, minimizing cross-talk and enabling precise analytical readouts.

Surface engineering strategies have been pivotal in enhancing both performance and applicability. Co-doping with elements such as boron has extended emission into the longer-wavelength region, increased oxidation stability, and improved selectivity for target analytes.^78^ These engineered N-MQDs have been successfully integrated into smart paper-based sensors and solid-state platforms, offering rapid detection times, high adsorption capacities, and portability, all while maintaining low limits of detection. The combination of fluorescence-based sensing with chemical capture not only provides a dual-function solution for environmental monitoring but also reduces assay complexity, reagent consumption, and operational costs, highlighting the economic advantages of these advanced nanomaterials.

Mechanistically, N-MQDs exploit a range of quenching and signal modulation phenomena to achieve dual-mode functionality. Static quenching and inner filter effects enable sensitive detection of heavy metals and metalloids, while ratiometric FRET facilitates simultaneous monitoring of metal ions and coordinating biomolecules.^79,80^ Reversible redox interactions, as demonstrated in the Cr(vi)/ascorbic acid system, further broaden the scope of detectable analytes and allow dynamic temporal monitoring.^81^ This mechanistic versatility underscores the ability of N-MQDs to respond predictably to diverse chemical environments, making them suitable for complex real-world samples, including wastewater, food matrices, and biological fluids.

Despite these achievements, several challenges and opportunities remain. First, while N-MQDs exhibit impressive selectivity and sensitivity, the long-term stability of the fluorescence signal under environmental conditions requires further investigation. Factors such as photobleaching, ionic strength variations, and surface fouling can influence signal fidelity. Second, the scalability and reproducibility of synthesis methods, including hydrothermal, ultrasonication, and inorganic base stripping approaches,^77,79^ must be optimized to support industrial and commercial applications. Current batch-to-batch variations in quantum yield and particle size can affect sensor performance, emphasizing the need for standardized, high-throughput production protocols.

In addition to these considerations, several practical aspects must be addressed to facilitate the real-world deployment of N-MQD-based sensing systems. Material stability remains a critical parameter, as prolonged exposure to environmental factors such as pH fluctuations, ionic strength variations, natural organic matter, and competing ions may alter surface states and consequently affect fluorescence response. Evaluating the operational stability of N-MQDs under realistic conditions, including long-term storage and repeated sensing cycles, is therefore essential. Another important factor is scalability. Although hydrothermal and exfoliation-based syntheses have demonstrated promising laboratory-scale yields, translating these approaches into reproducible, large-scale manufacturing processes remains challenging. Achieving uniform particle size distributions, consistent doping levels, and stable quantum yields across production batches will be necessary for reliable sensor fabrication. Furthermore, the performance of N-MQD probes in complex matrices such as wastewater, food extracts, or biological fluids requires systematic validation because matrix components can induce signal interference or non-specific adsorption.^48–79,83,84^ Finally, device integration poses additional challenges, including immobilization stability, signal calibration, and compatibility with portable detection platforms. Addressing these issues through standardized synthesis protocols, matrix-tolerant surface engineering, and robust device architectures will be crucial for bridging the gap between laboratory demonstrations and practical sensing technologies.

Moreover, integration with advanced analytical platforms represents a fertile area for innovation. The combination of N-MQDs with microfluidic devices, wearable sensors, and portable point-of-care systems could facilitate real-time monitoring of environmental pollutants, trace metals, and biomarkers, enabling proactive interventions. For instance, coupling NB-MQDs@PP paper-based platforms with automated filtration or digital readout systems could provide both qualitative and quantitative data on contaminant concentrations in near real-time.^78^ Similarly, incorporating N-MQDs into optical fiber or smartphone-based detection systems could democratize access to high-precision sensing tools, particularly in resource-limited regions.

In parallel with these technological developments, the industrial translation of N-MQDs-based sensing systems is beginning to gain attention. Several trends indicate a gradual movement from laboratory demonstrations toward scalable sensing technologies. First, the compatibility of N-MQDs with low-cost substrates such as cellulose paper, polymer films, and hydrogel matrices facilitates the development of disposable sensing devices suitable for large-scale environmental and food safety monitoring. Second, advances in scalable synthesis approaches, including continuous-flow hydrothermal processes and solution-phase exfoliation strategies, are improving production reproducibility and yield, which are critical parameters for industrial deployment. In addition, the integration of MQD-based fluorescence sensing with portable electronics, smartphone-assisted optical readers, and automated microfluidic sampling systems is creating practical pathways for field-deployable analytical tools.^73–76^ These developments are particularly relevant for sectors such as water quality surveillance, agricultural monitoring, and rapid on-site diagnostic testing. As manufacturing protocols become more standardized and device integration improves, N-MQDs-based dual-mode sensors are expected to transition from proof-of-concept studies toward commercially viable platforms capable of real-time, cost-effective chemical monitoring across diverse industrial environments.

From a mechanistic perspective, future research should explore rational surface functionalization for selective multiplexing, such as site-specific doping, post-synthetic modifications, and hybridization with other nanomaterials (*e.g.*, graphene oxide or metal–organic frameworks). These strategies could expand the excitation/emission spectrum, improve energy transfer efficiency, and fine-tune analyte specificity. Furthermore, advanced computational modeling and machine learning-guided design could predict the photophysical responses of engineered N-MQDs, enabling rapid optimization of dual-mode sensors prior to experimental validation. Environmental and economic considerations are also critical. N-MQDs present an inherently green and cost-effective alternative to traditional sensing platforms, particularly when integrated into recyclable or biodegradable substrates such as cellulose-based papers or hydrogels.^80^ Reducing the reliance on expensive or hazardous reagents, optimizing reaction conditions for energy efficiency, and exploring reusability strategies will further enhance the sustainability of these sensing technologies.

In conclusion, N-MQDs exemplify a next-generation platform for multiplexed dual-mode sensing, combining tunable photophysics, robust surface chemistry, and versatile integration potential. Their unique mechanisms, including static quenching, inner filter effects, FRET, and reversible redox interactions, provide a rich toolbox for encoding and decoding chemical information with high sensitivity and selectivity. Through continued advances in surface engineering, mechanistic understanding, and device integration, N-MQDs are poised to transform environmental monitoring, food safety analysis, and biomedical diagnostics. The path forward emphasizes scalable synthesis, mechanistic rationalization, and application-driven innovation, ensuring that these nanomaterials will remain at the forefront of dual-mode sensor research, offering both analytical precision and practical relevance for the next decade.

## Conflicts of interest

The authors declare that they have no known competing financial interests or personal relationships that could have appeared to influence the work reported in this paper.

## Data Availability

No primary research results, software or code have been included and no new data were generated or analysed as part of this review.
